# Tranexamic acid evokes pain by modulating neuronal excitability in the spinal dorsal horn

**DOI:** 10.1038/srep13458

**Published:** 2015-08-21

**Authors:** Nobuko Ohashi, Mika Sasaki, Masayuki Ohashi, Yoshinori Kamiya, Hiroshi Baba, Tatsuro Kohno

**Affiliations:** 1 Division of Anesthesiology, Niigata University Graduate School of Medical and Dental Sciences, 1-757 Asahimachi Dori, Chuo-Ku, Niigata City, 951-8510 Japan; 2 Division of Orthopedic Surgery, Department of Regenerative and Transplant Medicine, Niigata University Graduate School of Medical and Dental Sciences, 1-757 Asahimachi Dori, Chuo-Ku, Niigata City, 951-8510 Japan

## Abstract

Tranexamic acid (TXA) is an antifibrinolytic agent widely used to reduce blood loss during surgery. However, a serious adverse effect of TXA is seizure due to inhibition of γ-aminobutyric acid (GABA) and glycine receptors in cortical neurons. These receptors are also present in the spinal cord, and antagonism of these receptors in spinal dorsal horn neurons produces pain-related phenomena, such as allodynia and hyperalgesia, in experimental animals. Moreover, some patients who are injected intrathecally with TXA develop severe back pain. However, the effect of TXA on spinal dorsal horn neurons remain poorly understood. Here, we investigated the effects of TXA by using behavioral measures in rats and found that TXA produces behaviors indicative of spontaneous pain and mechanical allodynia. We then performed whole-cell patch-clamp experiments that showed that TXA inhibits GABA_A_ and glycine receptors in spinal dorsal horn neurons. Finally, we also showed that TXA facilitates activation of the extracellular signal-regulated kinase in the spinal cord. These results indicated that TXA produces pain by inhibiting GABA_A_ and glycine receptors in the spinal dorsal horn.

Tranexamic acid (TXA) is a synthetic lysine derivative that acts as an antifibrinolytic drug by inhibiting plasminogen activation and preventing fibrin degradation^1^,^2^,^3^,^4^,^5^. TXA is therefore commonly used to reduce perioperative blood loss, particularly during cardiac and orthopedic surgery^6^,^7^,^8^,^9^,^10^. However, several clinical studies reported that patients treated with TXA had a higher incidence of seizure after cardiopulmonary bypass^11^,^12^,^13^. TXA-associated seizure presumably results from inhibition of γ-aminobutyric acid (GABA) and glycine receptors in the brain, as the drug has been shown to inhibit these receptors in embryonic kidney cells and primary cortical neuron cultures^14^,^15^,^16^.

GABA and glycine are co-released by spinal dorsal horn neurons and are important in regulating sensory processing^17^,^18^,^19^,^20^. In addition, GABA and glycine receptors are abundant in the spinal cord, and antagonism of these receptors produces pain phenomena, such as allodynia and hyperalgesia^21^,^22^,^23^,^24^. Assuming that TXA acts as a GABA_A_ and glycine receptor antagonist in the spinal dorsal horn as well as in the cortex, we hypothesized that TXA evokes pain. Indeed, several clinical studies have reported that patients to whom TXA was accidentally injected intrathecally immediately complained of severe back pain^25^,^26^,^27^,^28^,^29^. Moreover, it has been reported that patients treated with TXA required higher doses of analgesics^30^,^31^. In addition, patients with menorrhagia who received TXA experienced a higher incidence of headache, abdominal pain, and back pain than did placebo-treated patients^32^. Thus, it seems possible that TXA impairs neuronal inhibition via blockade of GABA_A_ and glycine receptors and thereby produces pain at the spinal cord level. However, no previous studies have directly evaluated the effects of TXA on synaptic transmission in spinal dorsal horn neurons. In the present study, we therefore investigated whether clinical levels of TXA regulate synaptic transmission in the spinal cord, using behavioral measures, whole-cell patch-clamp techniques, and immunohistochemical analyses.

## Results

### TXA administration evokes spontaneous pain-like behavior and mechanical allodynia

**Figure 1:**
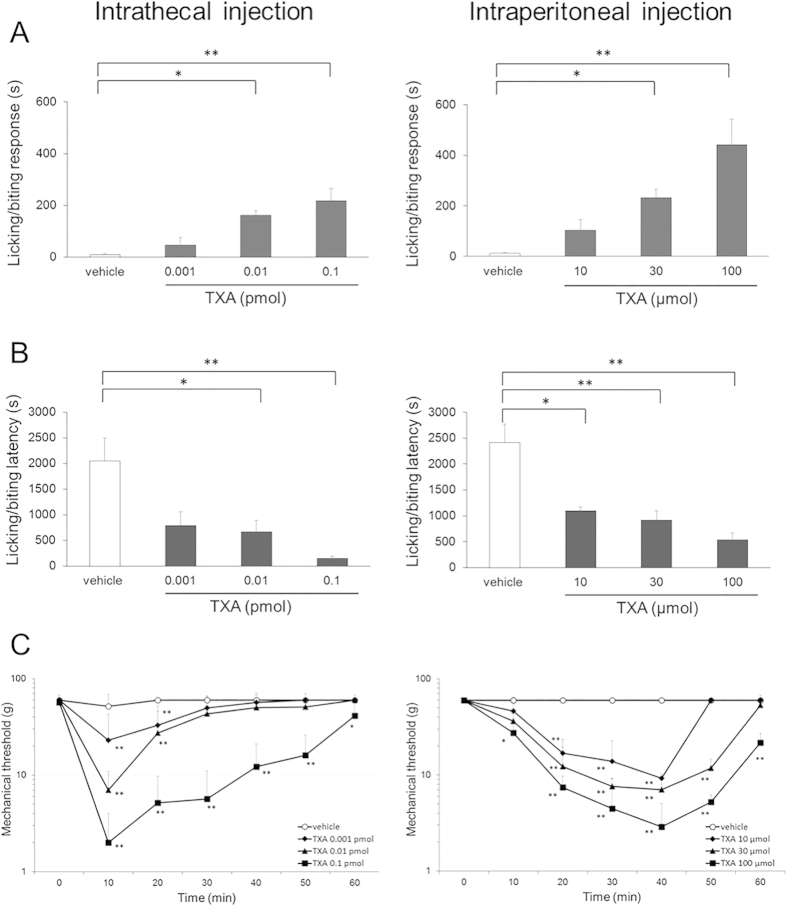
Assessment of behavioral response to intrathecal and intraperitoneal injection of tranexamic acid (TXA). (**A**) Total time devoted to licking/biting responses during a 60-min observation period is increased in a concentration-dependent manner by intrathecal or intraperitoneal injection of TXA. (**B**) The latency to the start of the behavior is shortened in a concentration-dependent manner by intrathecal or intraperitoneal injection of TXA. (**C**) Mechanical thresholds for paw withdrawal in response to von Frey stimulation are significantly reduced in a concentration-dependent manner by intrathecal or intraperitoneal injection of TXA. Concentrations of intrathecal or intraperitoneal injected TXA range from 0.001 to 0.1 pmol and from 10 to 100 μmol, respectively. The data are given as mean ± SD. In each dose group, *n *= 5; *P < 0.05, **P < 0.01 by one or two-way ANOVA.

To examine whether TXA evokes pain responses, we investigated the actions of TXA using behavioral measures, such as licking/biting, in rats. Total time devoted to licking/biting behaviors during the 60-min period following intrathecal or intraperitoneal injection of TXA increased in a concentration-dependent manner at doses from 0.001 to 0.1 pmol or from 10 to 100 μmol, respectively (each dose group: *n *= 5, *P *< 0.05 by one-way ANOVA, Fig. 1A). Similarly, the latency between TXA injection and the onset of licking/biting behaviors shortened in a concentration-dependent manner (each dose group: *n *= 5, *P *< 0.05 by one-way ANOVA, Fig. 1B).

The mechanical threshold for paw withdrawal during von Frey stimulation, which measures resistance to evoked pain, was significantly reduced by intrathecal or intraperitoneal injection of TXA in a concentration-dependent manner (each dose group; *n *= 5, *P *< 0.05 by two-way ANOVA, Fig. 1C). After intrathecal TXA injection, the sensitized response peaked at 10 min and then declined to baseline within 60 min. After intraperitoneal injection, the threshold decreased gradually to a peak at 40 min and lasted for more than 60 min. These results suggested that TXA produces behaviors indicative of spontaneous pain and mechanical allodynia following either intrathecal or intraperitoneal administration.

### TXA decreases the amplitude of miniature IPSCs (mIPSCs) without changing their frequency

**Figure 2:**
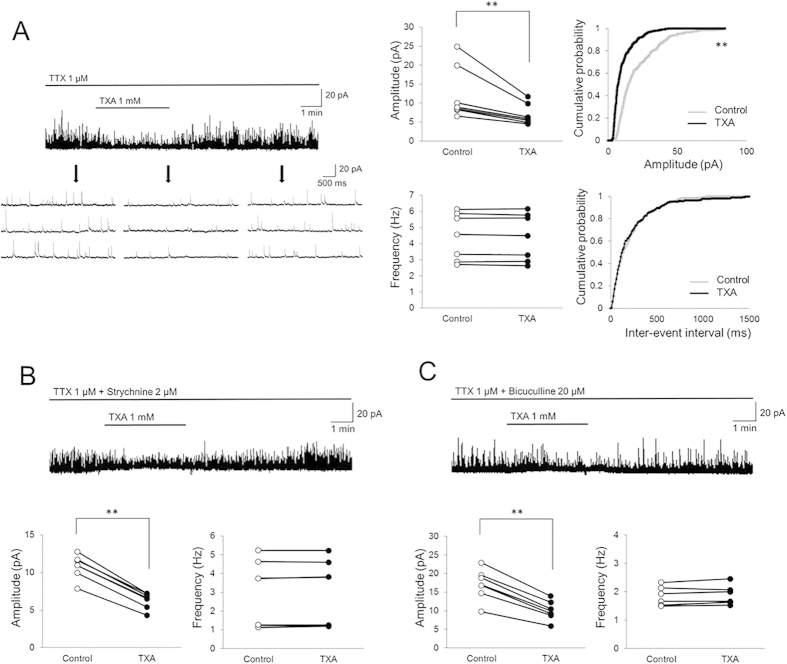
Tranexamic acid (TXA) decreases the amplitude of GABAergic and glycinergic mIPSCs without changes in frequency. (**A**) TXA (1 mM, 2 min) significantly decreases the amplitude of miniature inhibitory postsynaptic currents (mIPSCs) and shifts the cumulative distribution of the amplitudes to the left. In contrast, TXA has no effect on the mIPSC frequency or cumulative distribution of the inter-event intervals (*n *= 7). Downward arrows indicate outtakes of the top trace shown on an expanded timescale. Heavy horizontal bars show periods of drug application. (**B**) In the presence of strychnine (2 μM), a glycine receptor antagonist, TXA (1 mM, 2 min) significantly decreases GABAergic mIPSC amplitude. However, TXA has no effect on GABAergic mIPSC frequency (*n* = 7). (**C**) In the presence of bicuculline (20 μM), a GABA_A_ receptor antagonist, TXA (1 mM, 2 min) significantly decreases the amplitude of glycinergic mIPSCs. However, TXA has no effect on the frequency of glycinergic mIPSCs (*n *= 7). Holding potential* *= 0 mV for all recordings. **P < 0.01 by paired *t*-test.

We next investigated the mechanism of TXA action on SG neurons using the whole-cell patch-clamp technique. As it has been reported that TXA inhibits GABA and glycine receptors in the brain^14^,^15^,^16^, we examined the effect of TXA on inhibitory synaptic transmission pre- and/or postsynaptically. mIPSCs were isolated by adding TTX (1 μM) to the perfusate. In the presence of TXA (1 mM, 2 min), mean mIPSC amplitude decreased from 12.4 ± 7.1 to 7.0 ± 2.8 pA (59.9 ± 8.5% of control, *n *= 7, *P *< 0.01; Fig. 2A). Conversely, mean mIPSC frequency was unaffected by TXA (control, 4.4 ± 1.5; TXA, 4.4 ± 1.4 Hz, 99.4 ± 1.4% of control; *n *= 7, *P *= 0.98). Figure 2A (right panel) shows the effects of TXA on the cumulative distribution of the amplitudes and inter-event intervals of the mIPSCs. When compared to the control using the Kolmogorov-Smirnov test, superfusion with TXA increased the proportion of mIPSCs with significantly smaller amplitudes. However, TXA had no effect on the cumulative inter-event interval distribution of the mIPSCs over the recording period. These findings indicate that the TXA-induced decrease in inhibitory activity is postsynaptic in origin.

### TXA decreases the amplitude both of GABAergic and glycinergic mIPSCs without changing their frequency

To determine whether TXA acts on GABA or glycine receptors, we investigated the effects of TXA on GABAergic and glycinergic mIPSCs. GABAergic mIPSCs were isolated by adding TTX (1 μM) and the glycine receptor antagonist strychnine (2 μM) to the perfusate. In the presence of TXA, the mean GABAergic mIPSC amplitude decreased from 10.8 ± 1.6 to 6.3 ± 1.1 pA (58.2 ± 2.9% of control, *n *= 7, *P *< 0.01; Fig. 2B). Conversely, mean mIPSC frequency was unaffected by TXA (control, 3.0 ± 1.8; TXA, 3.0 ± 1.7 Hz, 101.2 ± 2.3% of control; *n *= 7, *P *= 0.98). Similarly, glycinergic mIPSCs were isolated by adding TTX and the GABA_A_ receptor antagonist bicuculline (20 μM) to the perfusate. In the presence of TXA, mean glycinergic mIPSC amplitude decreased from 17.1 ± 4.1 to 10.1 ± 2.6 pA (59.3 ± 2.8% of control, *n *= 7, *P *< 0.01; Fig. 2C). Conversely, mean mIPSC frequency was unaffected by TXA (control, 1.8 ± 0.3; TXA, 1.9 ± 0.3 Hz, 102.1 ± 3.2% of control; *n *= 7, *P *= 0.83). These results suggest that TXA inhibits postsynaptic GABA_A_ and glycine receptors located on SG neurons.

### TXA decreases the amplitude and integrated area of the current elicited by exogenous application of GABA or glycine

**Figure 3:**
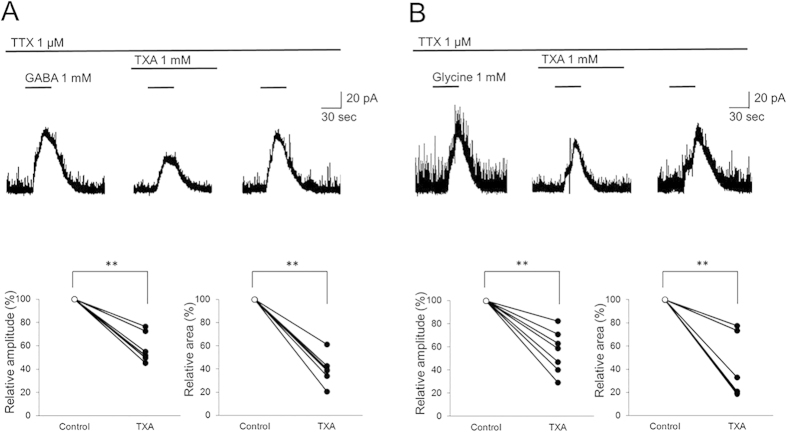
Tranexamic acid (TXA) inhibits the amplitude and decreases the integrated area of currents induced by exogenous GABA or glycine. (**A**) TXA (1 mM, 2 min) significantly decreases the amplitude and integrated area of the current elicited by exogenous application of GABA (1 mM, 30 s; *n *= 7). (**B**) TXA (1 mM, 2 min) significantly decreases the amplitude and integrated area of the current elicited by exogenous application of glycine (1 mM, 30 s; *n *= 7). Holding potential* *= 0 mV for all recordings. **P < 0.01 by paired *t*-test.

Next, we investigated the effects of TXA on the current induced by exogenously administered GABA or glycine (1 mM, 30 s) in SG neurons, in the presence of TTX to isolate postsynaptic actions. In the presence of TXA, the mean amplitude of the elicited GABA current decreased to 57.4 ± 12.2% of control, and the integrated area decreased to 40.0 ± 12.1% of control (*n *= 7, *P *< 0.01; Fig. 3A).

Similarly, the mean amplitude of the elicited glycine currents decreased to 55.8 ± 13.8% of control, and the integrated area decreased to 37.5 ± 14.6% of control (*n *= 7, *P *< 0.01; Fig. 3B), in the presence of TXA. These results reinforce the notion that TXA acts as an antagonist at postsynaptic GABA_A_ and glycine receptors.

**Figure 4:**
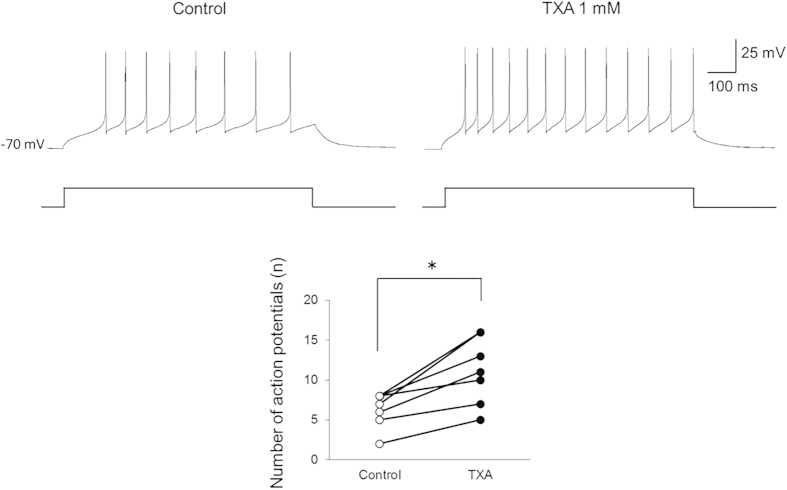
Tranexamic acid (TXA) increases the number of action potentials in dorsal horn neurons. TXA (1 mM) significantly increases the number of action potentials induced by current injection (100 pA, 1000 ms) in all recorded neurons (*n *= 7). *P < 0.05 by paired *t*-test.

Given that TXA decreases inhibition in SG neurons, it may also increase the potential for neuronal excitation. We therefore investigated action potential discharge activity in current-clamp mode. TXA significantly increased the number of action potentials from 6.9 ± 2.6 to 12.0 ± 4.8 (n* *= 7, *P *< 0.05 by paired *t*-test) when a depolarizing current was injected into the recorded neurons (Fig. 4A,B).

### TXA increases the frequency of spontaneous EPSCs (sEPSCs), but not of miniature EPSCs (mEPSCs), without changing their amplitude

**Figure 5:**
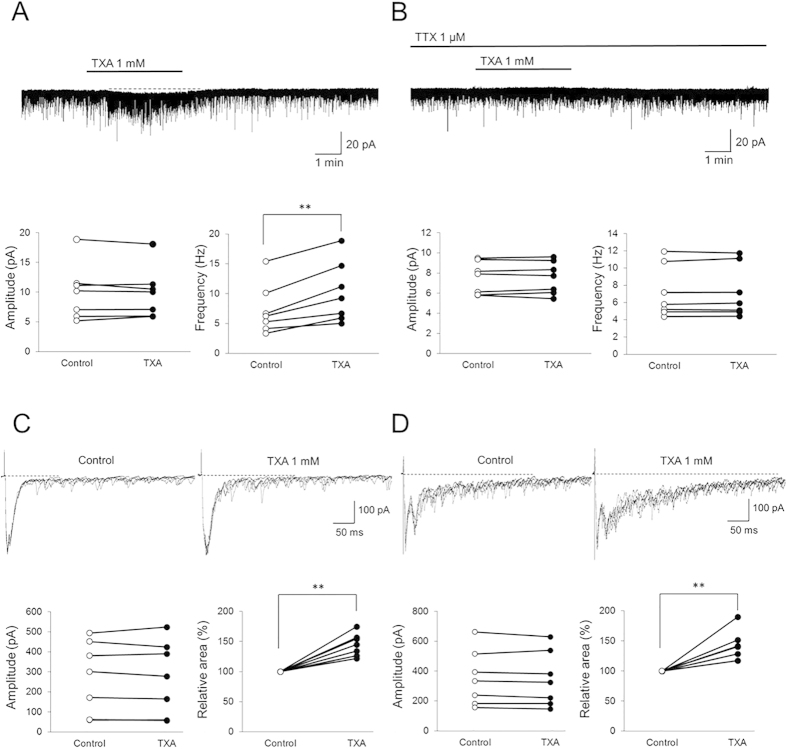
Tranexamic acid (TXA) facilitates excitatory glutamatergic transmission. (**A**) TXA (1 mM, 2 min) has no effect on the amplitude of spontaneous excitatory postsynaptic currents (sEPSCs). In contrast, TXA significantly increases sEPSC frequency (*n *= 7). Moreover, TXA induced inward currents (>5 pA) in all recorded neurons (*n *= 7). (**B**) TXA (1 mM, 2 min) has no effect on mEPSC amplitude or frequency (*n *= 7). (**C**) TXA has no effect on the amplitude of Aδ fiber-evoked monosynaptic EPSCs. In contrast, TXA significantly increases the integrated area of Aδ fiber-evoked polysynaptic EPSCs (*n *= 7). (**D**) TXA has no effect on the amplitude of C fiber-evoked monosynaptic EPSCs. In contrast, TXA significantly increases the integrated area of C fiber-evoked polysynaptic EPSCs (*n *= 7). Holding potential* *= −70 mV for all recordings. **P < 0.01 by paired *t*-test.

GABA_A_ and glycine receptor antagonists affect excitatory glutamatergic transmission^33^. Therefore, in the next set of experiments, we tested the effects of TXA on sEPSCs and mEPSCs, which represent glutamatergic transmission. In the presence of TXA, the mean sEPSC amplitude was not affected (control, 10.0 ± 4.7; TXA, 9.9 ± 4.3 pA, 100.6 ± 7.0% of control; *n *= 7, *P *= 0.97; Fig. 5A). However, the mean sEPSC frequency in the presence of TXA significantly increased from 7.3 ± 4.2 to 10.2 ± 5.1 Hz (144.0 ± 2.9% of control, *n *= 7, *P *< 0.01; Fig. 5A). Moreover, TXA induced an inward current (>5 pA) in all recording neurons (*n *= 7; Fig. 5A). The average peak amplitude of the TXA-induced inward current was 6.0 ± 8.1 pA. In contrast, neither the mean mEPSC amplitude (control, 7.5 ± 1.6; TXA, 7.5 ± 1.6 pA, 100.2 ± 3.6% of control; *n *= 7, *P *= 0.99; Fig. 5B) nor frequency (control, 7.2 ± 3.0; TXA, 7.2 ± 3.0 Hz, 100.5 ± 1.9% of control; *n *= 7, *P *= 0.98) was affected in the presence of TXA with mEPSCs isolated by adding TTX. Furthermore, the TXA-induced inward current was suppressed by adding TTX in all recording neurons (*n *= 7; Fig. 5B). These results suggest that TXA does not affect the presynaptic terminals of excitatory neurons, because TXA has no effect on the frequency of mEPSCs. In contrast, our results instead suggest that TXA acts on the somata of excitatory neurons, and by reducing inhibition facilitates glutamate release, which causes neuronal excitation, because TXA increases inward current and the frequency of sEPSCs but does not affect sEPSC/mEPSC amplitude.

### TXA increases the integrated area of polysynaptic EPSCs evoked by dorsal root stimulation

Primary afferent nerve fibers, such as Aδ and C fibers, terminate in the superficial layers of the spinal cord, particularly on SG neurons, and modulate pain information^34^,^35^. Therefore, we examined whether TXA acts on primary afferent terminals. Monosynaptic EPSCs evoked at Aδ fiber stimulation intensity can be mediated by Aδ fibers; however, polysynaptic EPSCs evoked at Aδ fiber stimulation intensity can be mediated by Aβ and Aδ fibers. Similarly, monosynaptic EPSCs evoked at C fiber stimulation intensity can be mediated by C fibers; however, polysynaptic EPSCs evoked at C fiber stimulation intensity can be mediated by Aβ, Aδ, and C fibers.

The amplitude of monosynaptic EPSCs evoked by Aδ fiber stimulation was not affected by TXA application (control, 274.5 ± 179.7; TXA, 271.0 ± 183.3 pA, 97.7 ± 5.7% of control; *n *= 7, *P *= 0.97; Fig. 5C). In contrast, TXA significantly increased the integrated area of polysynaptic EPSCs to 144.7 ± 18.8% of control (*n *= 7, *P *< 0.01). Similarly, the amplitude of monosynaptic EPSCs evoked by C fiber stimulation was not affected by TXA application (control, 355.0 ± 184.6; TXA, 347.0 ± 183.1 pA, 97.3 ± 4.0% of control; *n *= 7, *P *= 0.94; Fig. 5D), but the integrated area of polysynaptic EPSCs was significantly increased to 140.8 ± 25.0% of control (*n *= 7, *P *< 0.01).

These data indicated that TXA does not affect the primary afferent terminals of Aδ and C fibers, because it did not change the amplitude of monosynaptic Aδ or C fiber-mediated EPSCs. On the other hand, since TXA increased the integrated area of polysynaptic EPSCs evoked at Aδ or C fiber stimulation intensity, we concluded that TXA acts on excitatory interneurons and facilitates excitatory transmission from primary afferents indirectly.

### TXA enhances ERK activation

**Figure 6:**
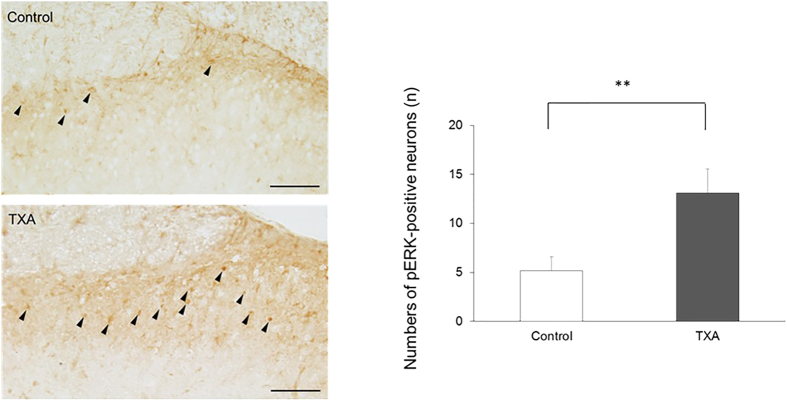
Tranexamic acid (TXA) enhances ERK activation in the superficial dorsal horn of the spinal cord. Under Krebs solution, few neurons are positive for pERK, an indicator of nociceptive stimulation, in slices of the superficial dorsal horn of the spinal cord (*n* = 7). In contrast, TXA (1 mM, 10 min) significantly increases the number of pERK-positive neurons (*n *= 7). ERK activation was quantified by counting the number of pERK-labeled neurons in the superficial dorsal horn in control and TXA groups. Filled arrowheads indicate pERK-positive neurons. Bar* *= 50 μm. The data are given as mean ± SD. **P < 0.01 by unpaired *t*-test.

To obtain spatial information on neuronal excitation in the spinal dorsal horn, we investigated the effect of TXA on neuronal pERK expression, which is indicative of pain. There were very few pERK-positive neurons in the superficial dorsal horn of control spinal cord slices when perfused with standard Krebs solution. However, following application of TXA to the perfusate for 10 min, the number of pERK-positive neurons significantly increased from 5.2 ± 1.4 to 13.1 ± 2.5 (*n *= 7, *P *< 0.01, Fig. 6). These results suggested that TXA enhances ERK activation.

## Discussion

We demonstrated that intrathecally or intraperitoneally administered TXA produces behaviors indicative of spontaneous pain and mechanical allodynia in rat in a concentration-dependent manner. To clarify the mechanism underlying these pain behaviors, we investigated the action of TXA on synaptic transmission in spinal dorsal horn neurons. We found that TXA alters excitatory and inhibitory activity at the spinal cord level, which could ultimately result in the experience of pain.

### TXA inhibits GABA_A_ and glycine receptors in spinal dorsal horn neurons

GABA and glycine are major inhibitory neurotransmitters in the central nervous system, and antagonism at their receptors causes seizure^36^,^37^,^38^. Therefore, TXA-associated seizure is presently attributed to inhibition of GABA and glycine receptors in the brain^14^,^15^,^16^. It is also known that GABA and glycine receptors are particularly abundant in the spinal dorsal horn and important for sensory processing^17^,^18^,^19^,^20^. Furthermore, spinal GABA_A_ and glycine receptors are co-localized on postsynaptic neurons, and these two neurotransmitters work together in sensory modulation^20^,^39^. Therefore, inhibition of spinal GABA_A_ and glycine receptors by TXA could dysregulate pain processing. In rodents, intrathecal administration of GABA_A_ or glycine receptor antagonists induces spontaneous pain-like biting and escape behaviors in response to light tactile stimulation^23^,^24^,^40^,^41^. Loss of GABAergic and glycinergic function may also manifest as allodynia and hyperalgesia in patients suffering from neuropathic pain. In this study, we demonstrated that TXA produces spontaneous and evoked pain in a concentration-dependent manner. We also demonstrated that TXA acts as a GABA_A_ and glycine receptor antagonist, as TXA decreased GABAergic and glycinergic mIPSC amplitude without affecting frequency. Moreover, TXA decreased the amplitudes and integrated areas of currents elicited by exogenous GABA or glycine application. Thus, it is plausible that the observed TXA-associated pain resulted from GABA_A_ and glycine receptor inhibition on spinal dorsal horn neurons.

Our findings also indicated that TXA enhances somatic excitability in SG neurons, presumably through direct inhibition of GABA and glycine receptors, as TXA significantly increased the number of action potentials induced in SG neurons by a standard depolarizing pulse. Taken together, our results suggest that TXA facilitates spinal dorsal horn neurons by inhibiting GABA and glycine receptors, resulting in pain of spinal cord origin.

### TXA facilitates excitatory transmission

Since TXA was found to modulate inhibitory transmission in spinal dorsal horn neurons, we next investigated whether TXA also affects excitatory transmission. TXA increased the frequency of sEPSC with inward current, but not that of mEPSC, without affecting amplitude, suggesting that TXA facilitates glutamatergic transmission by acting on excitatory SG interneurons. Primary afferent Aδ or C fibers reportedly modulate pain information by controlling excitatory and inhibitory interneuron activity^34^,^35^. As GABA_A_ and glycine receptors are thought to reside on these primary afferent terminals^18^,^42^, we investigated whether TXA acts on primary afferent terminals to modulate excitatory transmission. However, our results indicated that presynaptic inhibition of primary afferent terminals by TXA is not prominent, at least not in the fine afferent fibers of the superficial dorsal horn, because TXA did not affect the amplitude of the monosynaptic EPSCs evoked by stimulation of these fibers. The reason for the difference in results between previous studies and our study was most likely that presynaptic inhibition of primary afferent terminals is exerted exclusively on the large, myelinated Aα/β fibers in deep dorsal horn^43^,^44^. Indeed, one study reported that neither GABA_A_ nor glycine receptor agonist/antagonists affect monosynaptic EPSC amplitude in SG neurons^33^. In contrast, our findings suggest that TXA facilitates polysynaptic excitatory transmission because it increased the integrated area of polysynaptic EPSCs evoked by primary afferent stimulation. The abovementioned previous study reported that blockade of GABAergic and glycinergic inhibition facilitated polysynaptic excitatory synaptic transmission in SG neurons^33^. Our findings together with previous reports indicate that TXA enhances the excitability of excitatory interneurons via blockade of GABAergic and glycinergic postsynaptic inhibition, which facilitates excitatory transmission to the SG neurons indirectly.

**Figure 7:**
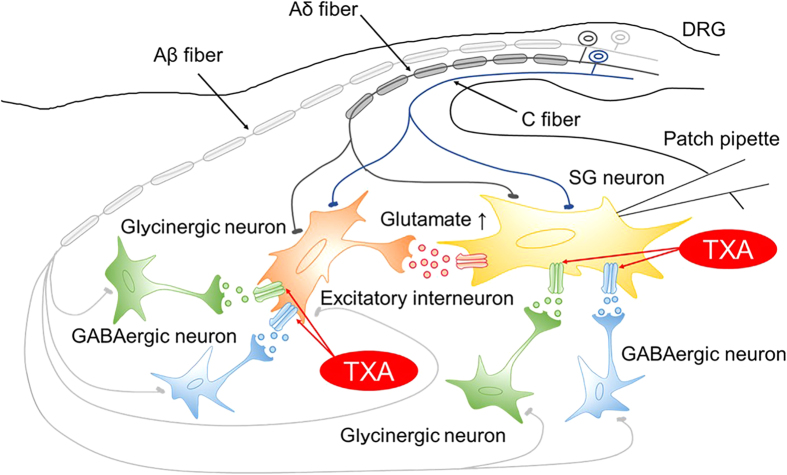
Model of the spinal dorsal horn circuit underlying the mechanism of tranexamic acid (TXA)-produced pain. TXA directly inhibits GABA and glycine receptors located on postsynaptic sites of the recorded SG neurons. TXA also inhibits GABA_A_ and glycine receptors located on postsynaptic sites on excitatory interneurons. This leads to increased glutamate release from the excitatory interneurons to the recorded SG neurons located postsynaptically, resulting in increased spontaneous activity. SG, substantia gelatinosa; DRG, dorsal root ganglion.

Given this, we propose the following model circuit for the underlying mechanism of TXA in the spinal dorsal horn (Fig. 7). TXA directly inhibits GABA_A_ and glycine receptors located on postsynaptic sites of the recorded SG neurons, resulting in increased neuronal excitability. In addition, TXA inhibits GABA_A_ and glycine receptors postsynaptically located on the excitatory interneurons, thus inducing excitability. Consequently, TXA leads to increased glutamate release from presynaptic excitatory interneurons to SG neurons. We propose that these mechanisms could produce pain.

### TXA enhances ERK activation, a marker of nociception

To confirm behavioral and electrophysiological evidence that TXA produces pain, we investigated the effect of TXA using immunostaining and demonstrated that TXA enhances ERK activation, a cellular marker of pain processing. ERK is the most intensively studied member of the family of mitogen-activated protein kinases^45^. ERK phosphorylation results from pain, especially in the superficial dorsal horn, and is implicated in the development of central sensitization^46^,^47^. We demonstrated that TXA increases pERK-positive neurons in the spinal cord, which is consistent with our behavioral data. These results suggested that TXA significantly excites a considerable number of dorsal horn neurons.

### TXA levels are within a clinically relevant range

The 1 mM concentration of TXA used in this study was chosen to imitate the effect of the clinical dose of TXA in cerebrospinal fluid. TXA reduces blood loss and transfusion requirements perioperatively at high doses of up to 100 mg/kg^48^,^49^,^50^,^51^. Following administration of 100 mg/kg TXA, plasma levels exceed 4 mM^52^, resulting in an estimated cerebrospinal fluid TXA concentration of 0.62–1.24 mM^12^,^53^,^54^. One previous study showed that TXA has an IC_50_ value of 7.1 ± 3.1 mM for recombinant GABA_A_ receptors in human embryonic kidney cells^14^, while another study reported IC_50_ values for TXA against GABA_A_ receptors of 0.76–0.84 mM in the murine amygdala^15^. The IC_50_ values for TXA against glycine receptors were 1.1–1.4 mM in primary cultures of embryonic murine cortical neurons^16^. Given that only 3% of TXA is bound to protein^55^, the concentration of TXA (1 mM) used in our study is thought to be within a clinically relevant range, and our study suggested that TXA acts directly on spinal neurons.

The 1 mM concentration of TXA was used in electrophysiological and immunohistochemical experiments because of clinical dose of TXA in cerebrospinal fluid according to the previous reports. However, 1 mM TXA intrathecally administered produced behaviors indicative of very severe spontaneous pain, which could not perform the assessment of behavioral response with von Frey filaments. Therefore, the concentration of TXA intrathecally administered is lower than that used in electrophysiological and immunohistochemical experiments. These results suggest that there is a possibility that TXA produces pain even with lower concentration of 1 mM.

In conclusion, we have demonstrated that TXA produces pain by inhibiting GABA_A_ and glycine receptors on spinal dorsal horn neurons at clinically relevant concentrations. Collectively, our data describe an underlying mechanism by which TXA can produce pain, and may contribute to the clinical management of TXA-associated pain.

## Materials and Methods

### Animals

Male Wistar rats (200–250 g) were used in all experiments. Animals were housed under a 12-h light/dark cycle with ad libitum access to food and water.

### Implantation of intrathecal catheter

For intrathecal drug administration, rats were implanted with a polyethylene PE-10 catheter, as previously described, with some modification^56^. Rats were anesthetized with 2–3% isoflurane, and a PE-10 polyethylene catheter was inserted rostrally into the lumbar enlargement through a mini-laminectomy at the L5 vertebra. The animals were allowed to recover for 3–6 days before experiments. Only animals without evidence of neurological dysfunction after catheter insertion were used for these studies.

### Assessment of behavioral response

Rats were acclimated to the experimental room for at least 30 min before injection of TXA. In the intrathecal group, TXA dissolved in normal saline was administered intrathecally through a catheter in 10-μL volume (from 0.001 to 0.1 pmol), followed by an injection of 10 μL of saline to flush the catheter. In the intraperitoneal group, TXA dissolved in normal saline was administered intraperitoneally with a 26-gauge needle in a volume of 1 mL (from 10 to 100 μmol).

Licking/biting behaviors were counted as a measure of spontaneous pain responses. Moreover, we investigated possible TXA-induced sensitivity to mechanical stimulation to assess evoked pain. The force threshold for paw withdrawal in response to probing with a series of calibrated von Frey filaments was therefore determined. Each filament was applied perpendicularly to the plantar surface of the paw of rats held in wire-mesh cages^57^. The withdrawal threshold was defined as the lowest force that evoked a clear withdrawal response at least twice in 10 applications, and was observed every 10 min for 60 min, beginning at TXA administration.

### Preparation of spinal cord slices

Rats were anesthetized with urethane (1.5 g/kg, intraperitoneal). A dorsal laminectomy was performed, and the lumbosacral segment of the spinal cord was removed. Rats were then euthanized immediately by exsanguination. The isolated spinal cords were placed in pre-oxygenated ice-cold Krebs solution. After severing all ventral and dorsal roots, except for the L4 dorsal root, the arachnoid membrane was removed. Each spinal cord was mounted on the metal stage of a microslicer (Linear Slicer PRO 7; Dosaka, Kyoto, Japan) and cut into 650-μm transverse slices with the L4 dorsal root attached. The slices were transferred to a recording chamber and perfused continuously with Krebs solution (10–15 mL/min) equilibrated with a 95% O_2_/5% CO_2_ gas mixture at 36 °C. The Krebs solution contained the following (in mM): NaCl 117, KCl 3.6, CaCl_2_ 2.5, MgCl_2_ 1.2, NaH_2_PO_4_ 1.2, NaHCO_3_ 25, and D-glucose 11.5.

### Patch clamp recording from dorsal horn neurons

Under a dissecting microscope with transmission illumination, lamina II of the dorsal horn was discernible as a relatively translucent band across the dorsal horn. Whole-cell patch-clamp recording of substantia gelatinosa (SG) neurons was conducted in voltage-clamp mode. After establishing the whole-cell configuration, voltage-clamped neurons were held at −70 mV for recording excitatory postsynaptic currents (EPSCs) and at 0 mV for recording inhibitory postsynaptic currents (IPSCs). The resistance of the patch pipette was 5–10 MΩ. The patch pipette solution contained the following (in mM): Cs_2_SO_4_ 110, CaCl_2_ 0.5, MgCl_2_ 2, EGTA 5, HEPES 5, tetraethylammonium 5, and ATP-Mg 5. Signals were amplified using an Axopatch 200B amplifier (Molecular Devices, Union City, CA) and were filtered at 2 kHz and digitized at 5 kHz. Data were collected and analyzed using the pCLAMP 10.4 software suite (Molecular Devices).

### Dorsal root stimulation

The L4 dorsal root was stimulated using a suction electrode. Stimulation was performed at 100 μA for 0.05 ms for Aδ fibers, and at 1000 μA for 0.5 ms for C fibers. Aδ fiber-evoked EPSCs were judged monosynaptic based on both their short and constant latencies and the absence of failures with repetitive stimulation at 20 Hz^35^. C fiber-evoked EPSCs were judged monosynaptic based on an absence of failures with low-frequency (1 Hz) repetitive stimulation. In contrast, polysynaptic EPSCs were recognized by their unreliable, variable latencies under such stimulation protocols.

### Action potential discharge activity

The effect of TXA (1 mM) on action potential discharge activity was examined in current-clamp mode using patch pipettes filled with potassium gluconate instead of cesium sulfate. The patch pipette solution contained the following (in mM): potassium gluconate 135, KCl 5, CaCl_2_ 0.5, MgCl_2_ 2, EGTA 5, and Mg-ATP 5. In response to a depolarizing current injection of 100 pA for 1000 ms, SG neurons exhibited a train of action potentials, and we investigated the effect of TXA on the number of action potential discharges.

### Immunohistochemistry

To measure the effect of TXA on spinal dorsal horn with phosphorylated extracellular signal-regulated kinase (pERK), spinal cord slices were perfused with Krebs solution for at least 3 h before TXA application, after which TXA (1 mM) was applied in the perfusate for 10 min. After drug treatment, the slices were fixed in 4% paraformaldehyde for 60 min, equilibrated with sucrose overnight, cut on a cryostat at a thickness of 16 μm, and mounted on slides. Sections were incubated with rabbit anti-pERK1/2 antibody (Cell Signaling Technology, Danvers, MA; 1:1,000) for 2 days at 4 °C. The sections were then incubated with biotinylated anti-rabbit secondary antibody (Vector Laboratories, Burlingame, CA; 1:400) for 4 h at room temperature. Sections were then processed with a Vectastain ABC system kit (Vector Laboratories) following the manufacturer’s instructions. To determine the mean number of pERK-positive neurons in the superficial laminae (I–II), at least five nonadjacent sections were randomly selected, and cells were counted under a microscope-digital camera system (Nikon, Tokyo, Japan).

### Drug application

The drugs used in this study were TXA, bicuculline, strychnine, GABA, glycine (all from Sigma–Aldrich, St. Louis, MO) and tetrodotoxin (TTX; from Wako, Osaka, Japan). Bicuculline was first dissolved in DMSO at a 1,000 times the final concentration for storage, and the other drugs were first dissolved in distilled water at a 1,000 times the final concentrations for storage. These stock solutions were diluted to the final concentration with Krebs solution immediately before use.

### Study approval

All animal experiments were conducted in accordance with international guidelines on the ethical use of animals, and all efforts were made to minimize the amount of pain or discomfort experienced by the animals. Animal housing and surgical procedures were approved by the Institutional Animal Care and Use Committee of Niigata University Graduate School of Medical and Dental Science (Approval No. 342-7).

### Statistical Analysis

Data are expressed as mean ± SD. Statistical significance was defined as *P *< 0.05 by a Student’s paired or unpaired *t*-test, one or two-way ANOVA followed by a Bonferroni post hoc test for multiple comparisons. The StatView program 5 (SAS Institute, Cary, NC, USA) was used for statistical analysis.

## Additional Information

**How to cite this article**: Ohashi, N. *et al.* Tranexamic acid evokes pain by modulating neuronal excitability in the spinal dorsal horn. *Sci. Rep.* **5**, 13458; doi: 10.1038/srep13458 (2015).
